# Nutrition in Patients with Idiopathic Pulmonary Fibrosis: Critical Issues Analysis and Future Research Directions

**DOI:** 10.3390/nu12041131

**Published:** 2020-04-17

**Authors:** Paola Faverio, Marialuisa Bocchino, Antonella Caminati, Alessia Fumagalli, Monica Gasbarra, Paola Iovino, Alessandra Petruzzi, Luca Scalfi, Alfredo Sebastiani, Anna Agnese Stanziola, Alessandro Sanduzzi

**Affiliations:** 1 School of Medicine and Surgery, University of Milano-Bicocca, 20900 Monza, Italy; paola.faverio@gmail.com; 2 Respiratory Unit, San Gerardo Hospital, ASST Monza, 20900 Monza, Italy; 3 Section of Respiratory Diseases, Department of Clinical Medicine and Surgery, Federico II University, 80131 Naples, Italy; marialuisa.bocchino@unina.it; 4 Unit of Pneumology and Respiratory Semi-Intensive Care Unit, Respiratory Pathophysiology and Pulmonary Hemodynamics Service, San Giuseppe Hospital—MultiMedica IRCCS, 20123 Milan, Italy; lafitta@libero.it; 5 Unit of Pulmonary Rehabilitation, IRCCS INRCA (Italian National Research Centre on Aging), 23880 Casatenovo, Italy; a.fumagalli@inrca.it; 6 Association “Un Respiro di Speranza” in Collaboration with the Department of Pulmonary Diseases of San Camillo-Forlanini Hospital, 00152 Rome, Italy; monicagasbarra@libero.it; 7 Gastrointestinal Unit, Department of Medicine, Surgery and Dentistry “Scuola Medica Salernitana”, University of Salerno, 84081 Salerno, Italy; piovino@unisa.it; 8 MEDICA—Editoria e Diffusione Scientifica, 20124 Milan, Italy; 9 Applied Nutrition and Health-Related Fitness, Department of Public Health, School of Medicine, Federico II University, 80131 Naples, Italy; scalfi@unina.it; 10 Department of Respiratory Diseases, San Camillo-Forlanini Hospital, 00152 Rome, Italy; alfredosebastiani23@gmail.com; 11 Section of Respiratory Disease, Department of Clinical Medicine and Surgery, Monaldi Hospital, Federico II University, 80131 Naples, Italy; annastanziola@libero.it (A.A.S.); sanduzzi@unina.it (A.S.)

**Keywords:** idiopathic pulmonary fibrosis, nutrition abnormalities, nutritional assessment, rehabilitation

## Abstract

In idiopathic pulmonary fibrosis (IPF), several factors may have a negative impact on the nutritional status, including an increased respiratory muscles load, release of inflammation mediators, the coexistence of hypoxemia, and physical inactivity. Nutritional abnormalities also have an impact on IPF clinical outcomes. Given the relevance of nutritional status in IPF patients, we sought to focus on some critical issues, highlighting what is known and what should be further learned about these issues. We revised scientific literature published between 1995 and August 2019 by searching on Medline/PubMed and EMBASE databases including observational and interventional studies. We conducted a narrative review on nutritional assessment in IPF, underlining the importance of nutritional evaluation not only in the diagnostic process, but also during follow-up. We also highlighted the need to keep a high level of attention on cardiovascular comorbidities. We also focused on current clinical treatment in IPF with Nintedanib and Pirfenidone and management of gastrointestinal adverse events, such as diarrhea, induced by these antifibrotic drugs. Finally, we concentrated on the importance of pulmonary rehabilitation program, including nutritional assessment, education and behavioral change, and psychological support among its essential components. More attention should be devoted to the assessment of the undernutrition and overnutrition, as well as of muscle strength and physical performance in IPF patients, taking also into account that an adequate clinical management of gastrointestinal complications makes IPF drug treatments more feasible.

## 1. Introduction

In recent years, nutritional status has increasingly gained attention in the evaluation of patients with chronic respiratory diseases since their clinical course is often characterized by progressive weight loss and reduction of muscle mass [1,2,3]. 

Idiopathic pulmonary fibrosis (IPF) is a fibrosing interstitial lung disease of unknown etiology characterized by rapid progression and poor prognosis [4,5].

**Figure 1:**
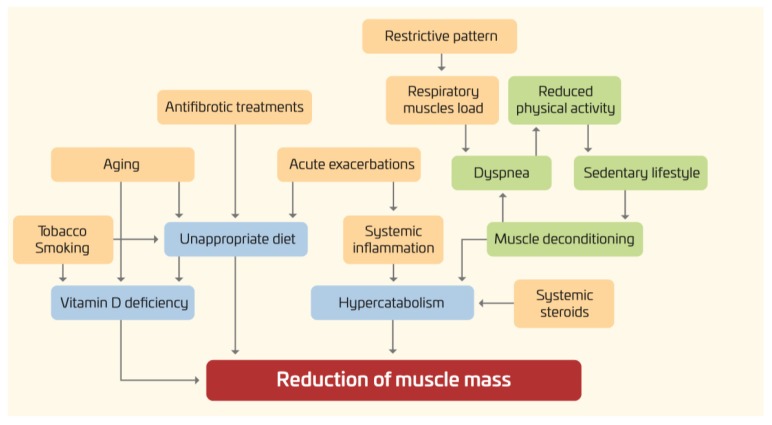
Nutritional disorders in IPF: pathogenesis.

As shown in Figure 1, in IPF, several factors may have a negative impact on the nutritional status of IPF patients, including an increased respiratory muscles load, release of inflammation mediators, hypoxemia, and physical inactivity [6,7,8,9].

Furthermore, given the rapidly progressive nature of the disease, with a median survival time of only two to five years, all these factors often develop and worsen in a short period of time.

It is worth noting that not only may the “downward spiral” of the underlying pulmonary disease affect the nutritional status, but also nutritional abnormalities may have an impact on clinical outcomes. For instance, lower body mass index (BMI) and body weight loss have been associated with increased mortality [10,11,12]. Conversely, the effects of obesity and metabolic syndrome are less known.

Given the relevance of evaluating nutritional status in IPF patients, a topic which remains still largely understudied, we sought to focus on some critical issues, highlighting what is known and what should be further learned about these issues.

## 2. Materials and Methods

We revised scientific literature published between 1995 and August 2019 by searching on Medline/PubMed, EMBASE, and clinicaltrials.gov databases including observational and interventional studies. Keywords used for each database are reported in Box 1.

Box 1 **Selected keywords to perform the research.**

Nutritional status AND (IPF OR acute exacerbation of IPF), nutritional assessment AND (IPF OR acute exacerbation of IPF), nutrition AND (IPF OR acute exacerbation of IPF), body mass index AND (IPF OR acute exacerbation of IPF), nutritional interventions AND (IPF OR acute exacerbation of IPF), dietary habits AND (IPF OR acute exacerbation of IPF), body composition AND (IPF OR acute exacerbation of IPF), metabolic rate and (IPF OR acute exacerbation of IPF), body weight AND (IPF OR acute exacerbation of IPF), weight loss AND (IPF OR acute exacerbation of IPF), weight gain AND (IPF OR acute exacerbation of IPF), malnutrition AND (IPF OR acute exacerbation of IPF), overweight AND (IPF OR acute exacerbation of IPF), obesity AND (IPF OR acute exacerbation of IPF), cardiovascular comorbidities AND IPF, cardiovascular risk AND IPF, dyslipidemia AND IPF, metabolic syndrome AND IPF, diabetes mellitus AND IPF, gastroesophageal reflux disease AND IPF, nintedanib AND (gastrointestinal events OR diarrhea OR nausea OR vomiting OR dyspepsia OR anorexia), pirfenidone AND (gastrointestinal events OR diarrhea OR nausea OR vomiting OR dyspepsia OR anorexia), IPF AND pulmonary rehabilitation.

Studies targeting children and conference abstracts were excluded. In addition, given the aim of the present review, we included only studies on IPF excluding those on other interstitial lung diseases (ILDs). Topics and structure of the review were discussed and approved by the expert panel. Reviewing main topics were based on the author’s expert view on the current status of the field under discussion.

## 3. Results and Discussion

### 3.1. Assessment of Nutritional Status in IPF Patients

Specific nutritional counseling and dietary advices for IPF patients are not yet available, with the exception of dietary recommendations to reduce the side effects of antifibrotic treatments (see the following sections). The implementation of a nutrition care process may be useful especially in those patients with IPF who are at risk or already malnourished [13]. According to the ESPEN guidelines [13], malnutrition (i.e., undernutrition) can be defined as “a state resulting from lack of intake or uptake of nutrition that leads to altered body composition (decreased fat free mass) and body cell mass leading to diminished physical and mental function and impaired clinical outcome from disease”. Disease-related malnutrition can arise from a number of reasons, for instance the inability to eat (for instance, as a result of anorexia, nausea, and vomiting), impaired absorption, altered metabolism, and hypercatabolism. The diagnosis of malnutrition (undernutrition) may be reached according to international criteria [13] or based on authors’ choices. The term overnutrition includes both overweight and obesity.

From a practical point of view, nutritional status may be assessed by evaluating nutrient intakes, energy expenditure, body composition, laboratory data, and body functions. Overall, only a few studies (in most cases, cross-sectional) have so far evaluated the nutritional status of IPF patients.

To the best of our knowledge, there are no consistent results available on nutrient intakes in IPF. Two studies carried out in Japan [14,15] showed that the consumption of fruit was associated with a reduced risk of IPF, whereas the opposite was true for meat and saturated fatty acids.

Main anthropometric data were reported in many papers. Mean BMI was around 26–28 kg/m^2^ in most studies from Western Countries [8,10,16,17,18,19,20,21] and lower (23–24 kg/m^2^) in those carried out in Japan and South Korea [11,22,23,24,25] (Table 1).

Thus, a high proportion of overweight/obese patients (overnutrition) may be expected [17,21]. As a matter of fact, in terms of phenotype, the prevalence of obese patients has been reported in two studies to be 34% and 46% [10,21]. On the other hand, the prevalence of underweight patients was found low (4%), but of clinical significance, in a recent study on French IPF patients [18], whereas it was substantially higher (>20%) in patients recruited in Japan [24]. As far as body composition is concerned, two studies found low fat-free mass (strongly suggesting malnutrition) in a consistent proportion of IPF patients [18,24]. The study by Jouneau et al. also reported low values of mid-arm circumference in nearly one-third of cases [18]. Interestingly, a reduction of pectoralis muscle area (no difference in BMI) was specifically observed in IPF patients with frailty syndrome [21]. Finally, it should be stressed that fat-free mass is also a significant independent predictor of survival in IPF patients [24]. With regard to vitamin status, a recent study showed that patients with IPF exhibited low serum vitamin D concentrations and such deficiency correlated with all-cause mortality, suggesting the role of vitamin D as prognostic factor and therapeutic target [26]. Overall, it should be noted that there are no studies identifying malnutrition and/or sarcopenia, as formal diagnoses, using to international criteria such as those proposed by ESPEN [13] or the 2019 EWGSOP Consensus [27], respectively.

The assessment of health-related physical fitness provides relevant information on body functions by assessing muscle strength, physical performance, and exercise tolerance. There is a small amount of literature on this issue for IPF patients. An inverse association between handgrip strength and age or dyspnea was observed [8,28]. Gait speed was inversely correlated with gender, age, and GAP index (a staging system based on gender, age and physiology, i.e., pulmonary function) and improved with pulmonary rehabilitation [20]. Furthermore, it was also an independent predictor of mortality [17]. A greater six-minute walk distance (6MWD) was associated with a higher diffusion capacity of the lung for carbon monoxide (DLCO) and forced vital capacity (FVC), lower dyspnea, better quality of life, and lower mortality [29].

### 3.2. Nutritional Follow-Up in IPF Patients

Considering that there are no guidelines about the nutritional follow-up in IPF patients, in our opinion, nutritional status should be re-evaluated at regular intervals in all IPF patients with regard to formal diagnosis, weight loss, and nutritional problems. According to the best clinical practice, the periodic evaluation should consider [30] dietary intake and nutrition-focused medical history; nutrition-related physical signs and symptoms; body composition [31]; basal metabolic rate [32]; laboratory tests [11]; functional tests [24]; and physical activity level [12].

Previous studies have reported that body weight changes with time have a strong correlation with clinical outcomes in several diseases [30,31]. Body weight loss is recognized as a common complication of IPF [32]. In fact, there is evidence that body weight loss is an independent predictive factor of reduced survival in IPF [11] and a decrease in BMI >5% per year are associated with an increased mortality even in patients with an FVC decline <10% [24]. Body weight loss is not affected by BMI values at baseline, suggesting that a single-point measurement is not enough to evaluate the weight of the status of IPF patients [11]. A recently published retrospective study confirmed that weight loss is common in patients with ILD and it is associated with an increased risk of mortality in patients with IPF. The authors suggest that weight loss may also serve as a longitudinal marker of disease progression together with functional evaluation [12]. Body weight loss may be due to various factors such as chronic inflammation, oxidative stress, reduced food intake (due to loss of appetite), and may be associated with disease progression [33]. In addition, in IPF, as for other chronic disease as chronic obstructive pulmonary disease (COPD), the cause of nutritional abnormalities is multifactorial. The different relevance, if any, of nutritional change in IPF patients in comparison with other chronic lung disease has not been studied so far.

Finally, as far as body composition is concerned, changes in muscle mass body composition, i.e., a reduced cross-sectional area of erector-spinae muscles, are associated with a worse prognosis in IPF patients [34].

### 3.3. Nutrition and Acute Exacerbation of IPF

Acute exacerbation is characterized by acute respiratory worsening, being a severe event in the course of IPF and leading to high mortality [35]. In this situation, patients with IPF are almost always hospitalized, with no data so far available on their nutritional status. A basal evaluation of nutritional status in hospitalized IPF patients may be measured with serological markers such as total proteins and total cholesterol level. However, there are no data about nutritional evaluation in this condition. Indeed, to produce some comments, we can examine and ponder available evidence on seriously ill patients hospitalized for other diseases.

Malnutrition is a highly prevalent consequence of hospitalization, especially in critically ill patients admitted to intensive care units (ICU) [36]. Malnutrition can be the result of both hypermetabolism, and inadequate intakes of energy and protein [37,38]. During IPF exacerbation, a greater reduction in physical activity, impairment in gas exchange and oxygenation, a systemic inflammatory condition, and a worse nutritional balance may be observed. Inflammation helps accelerate the degradation of muscle proteins. Immobilization is another important factor in muscle myopathy among ICU patients [39].

Nutrient deficiency has been correlated with a prolonged length of ICU/hospital stay and is strongly associated with increased morbidity and mortality among critically ill patients [36,37]. Conversely, medical nutritional therapy may reduce morbidity, mortality, and length of ICU stay [40]. Nutrition support in the ICU/hospital can impact favorably the development of complications, the modulation of the immune response, and the length of stay, resulting in improved outcomes [41,42].

### 3.4. Cardiovascular Risk, Metabolic Syndrome, and Obesity

Cardiovascular comorbidities, particularly coronary artery disease and arrhythmias, have been frequently observed in patients with IPF [43,44,45]. Such comorbidities may increase mortality risk [46]. Risk factors for cardiovascular diseases in IPF patients may include (1) fibrosing pathogenetic pathways shared with IPF that still largely remain to be explored; (2) unhealthy lifestyles and eating habits, such as low physical activity level, dyslipidemia, metabolic syndrome, systemic hypertension, and smoking history [47]. To note, multiple post-hoc analyses evaluated the effect of cardiovascular drugs on IPF outcomes: angiotensin-converting enzyme inhibitor (ACEi) and statin use, but not angiotensin II receptor blocker (ARB) administration, was associated with slower disease progression [48,49].

Prevalence of diabetes mellitus also appears higher than in general population, even after the exclusion of individuals treated with systemic corticosteroid therapy. However, the prognostic significance of such finding is unknown [46]. Metformin, a well-known antidiabetic drug, has been recently explored as antifibrotic agent in the mouse model of bleomycin-induced pulmonary fibrosis with positive results. More specifically, metformin significantly reduced the expression of multiple profibrotic proteins and restored mitochondrial biogenesis accelerating lung fibrosis resolution [50,51,52]. Moreover, Spagnolo et al. [53] performed a post-hoc analysis to assess the effect of metformin on IPF outcomes including mortality, hospitalization, FVC, and 6MWD decline and found no differences in disease progression or other outcomes at 1 year.

As already mentioned, observational studies reporting BMI in patients with IPF showed a presence of overweight or obesity in the majority of patients [8,11,18].

Although in IPF patients lower BMI and weight loss show a negative impact on disease outcomes more than overweight and obesity (see above), these latter issues may have a major impact in advanced stages as demonstrated by the fact that obesity is associated with an increased mortality risk in patients receiving bilateral lung transplantation and that obese patients may not be eligible for transplant [54]. Interestingly, the prevalence of hypothyroidism was observed to be higher and an independent predictor of mortality in IPF patients as compared to the general population, and it may also be a cause of overweight and obesity; therefore, in patients with such conditions, it is important to screen for dysthyroidism [55].

### 3.5. Gastrointestinal Aspects: Gastroesophageal Reflux Disease

A recent meta-analysis confirmed that gastroesophageal reflux disease (GERD) and IPF may be associated with each other; however, this association was found in case-control studies with smoking being a possible major confounder [56]. The causal association would still have to be clearly demonstrated. The current paradigm of GERD diagnosis hinges on the identification of esophageal mucosal lesions or troublesome symptoms caused by GERD [57]. A putative GERD diagnosis is reinforced by a favorable response to proton pump inhibitor (PPI) therapy [58]. Standardized questionnaires may be useful in the diagnostic process [59]. Indications for further testing include treatment failure, diagnostic uncertainty, and treating (or preventing) complications of GERD. However, diagnostic testing may or may not support the initial diagnosis, as the criteria defining GERD are specific to each testing modality [60]. The optimal diagnostic strategy to test for GERD in patients with IPF is uncertain. The benefit of antacid medication on IPF progression is unclear because there are conflicting and low-quality data. Spanish and German guidelines for IPF do not recommend antacids treatment of IPF patients for their primary disease underlying the need of further studies [61,62]. Conversely, the recommendations of the clinical practice guidelines suggest a regular antacid treatment for patients with IPF due to a possible (and yet unproven) increase in lung function and survival, and the low cost of therapy [63]. However, a potential risk for increased infections should be taken into account [64].

Although dietary intervention might play a role in effective treatment and management of GERD [65] and reflux itself might adversely affect nutritional status in chronic debilitating condition [66], to the best of our knowledge, there are no studies who address the complex interplay between IPF, nutritional status, and GERD. Further studies are needed to evaluate the effective role of antacids on IPF progression.

### 3.6. Adverse Events of New Antifibrotic Drugs

The two currently available anti-fibrotic drugs, nintedanib and pirfenidone (PFD), have offered a therapeutic chance to IPF patients. However, as reported below, both of them cause adverse events that may interfere with food intake and absorption, potentially leading to weight loss and malnutrition. To the best of our knowledge, the relation between nutrition and anti-fibrotic therapies has not been carefully investigated and needs special attention. There is evidence that a weight loss > 5% occurs in about 20% of IPF patients, even in the absence of anti-fibrotic treatments [11]. Moreover, both weight loss and a decrease in BMI have been associated with a worse prognosis [11,67,68]. Recently, Perelas et al. reported that the choice of anti-fibrotic medication along with disease severity predicts weight loss. In particular, a higher proportion (61%) of patients taking nintedanib compared to those taking PFD (30%) had a clinically significant weight loss after one year of uninterrupted treatment [69].

#### 3.6.1. Nintedanib

In IPF patients, the treatment with Nintedanib was not associated with serious adverse events (data from INPULSIS and INPULSIS ON trials), but was associated with increased gastrointestinal events, particularly diarrhea (62.4% in treatment group vs. 18.4% in placebo group). These episodes were mild-to-moderate and led to a low 4.4% incidence of discontinuation in the clinical trial [70]. Additionally, diarrhea tended to ameliorate after the first months of treatment. In INPULSIS, 44% of diarrhea cases occurred within the first month of treatment and 67% within the first three months. INPULSIS studies were extended in the INPULSIS ON, and data from INPULSIS ON suggested that a correct clinical management can reduce adverse events in IPF patients; in fact, INPULSIS placebo patients, which received Nintedanib in the subsequent INPULSIS ON, experienced a lower rate of side effects. Specifically, the INPULSIS studies showed an event rate for diarrhea of 112.6 (per 100 PEY) vs. 25.6 of placebo, while the INPULSIS ON reported only 62.5 events in the nintedanib group and 73.7 events in patients who began treatment (formerly placebo arm) [71]. Real life data emerging about nintedanib use in IPF patient confirmed the efficacy and safety observed in the clinical trials.

Gastrointestinal adverse events can be managed by reduction to 100 mg twice daily of Nintedanib dose or temporary suspension as needed. For diarrhea control, concomitant usage of loperamide [72,73] as well as appropriate nutrition have been suggested without negative effects on antifibrotic efficacy [74].

Moreover, Ikeda et al. [75] observed an increased risk of hepatotoxicity in Japanese patients with low surface area treated with nintedanib 150 mg twice daily. In 8 out of 10 patients, this adverse event did not develop again after resuming a reduced dose of 100 mg twice daily. These preliminary data suggest the importance of paying attention to patients with a small build, considering dose reduction if needed.

#### 3.6.2. Pirfenidone

A retrospective comprehensive analysis of safety outcomes of treatment with pirfenidone (PFD) in 1299 IPF patients from five clinical trials (including two studies with no control group, and the exclusion of patients with selected co-morbidities like renal failure and hepatic impairment) has shown that overall PFD was well tolerated over a 9.9 years follow-up. Mild to moderate nausea (37.6%), diarrhea (28.1%), dyspepsia (18.4%) and vomiting (15.9%) were the most frequent adverse events [76]. PFD has also been shown to be well tolerated (and equally efficacious) in patients with more advanced IPF without an increased risk of discontinuation due to therapy-related adverse events [77,78,79].

In real-life studies, anorexia and gastrointestinal disturbance are the most common reasons for cessation of PFD [80,81]. Ikeda et al. [25] recently reported that PFD was associated with anorexia, weight loss, and dyspepsia, respectively, in the 63.3%, 56.7%, and 16.7% of 30 IPF patients who stopped therapy. Moreover, anorexia and weight loss increased the rate of early termination (within six months) of those patients treated with PFD; due to these issues, such patients switched to nintedanib. However, the overall safety profile of PFD seems to be quite good [82,83]. Certainly, temporary dose modification or drug interruption may help the management of adverse events with patients counselling being encouraged [84]. Safety data of combination therapy with pirfenidone and nintedanib suggest that their use for 24 weeks was well tolerated by 89 IPF patients. However, the majority of them (74, mean baseline BMI 28.6 kg/m^2^) had 418 treatment-related adverse events with nausea, vomiting, and diarrhea being the most common [85]. In order to help patients with nausea and vomiting, it should be suggested to eat small snacks throughout the day, avoid large meals, or eat food that is cold or at room temperature (see below).

### 3.7. Management of Antifibrotic Drugs-Induced Diarrhea

The benefit of specific dietary recommendations other than those for oral hydration has not been well established in controlled trials.

However, a diet can play a role in treating diarrhea avoiding foods that are high in fat, high in fiber, milk and dairy products (since lactase deficiency may be induced by mucosal injury), spicy foods, alcohol, caffeine-containing products, some fruit juices (e.g., prune juice, orange juice) [86]. Main recommendations for gastrointestinal adverse events including diarrhea, nausea and vomiting, and appetite loss are summarized in Table 2.

### 3.8. Pulmonary Rehabilitation

Pulmonary rehabilitation (PR) has been widely used in the treatment of chronic respiratory diseases, playing a fundamental role in improving exercise capacity, health-related quality of life, and breathlessness.

PR is one of the most effective nonpharmacological treatments, and it is recommended by guidelines for patients with IPF, even if the evidence is still inconclusive.

As summarized in in the review performed by Xuequing et al., pulmonary function was evaluated only in three studies, showing a significant positive effect of PR on FVC in IPF patients, with no statistically significant improvement was reported for DLCO [87].

A randomized controlled trial, conducted by Perez-Bogerd et al. showed that exercise capacity, health status, and muscle force improved significantly after PR, maintaining such results after one year [88]. In addition, Dowman et al. found that successful adherence to exercise progression maximizes the benefits [89].

Moreover, Chehere et al. reported a 53% non-responder rate, related to the severity of exercise-induced desaturation and the impairment of DLCO before PR [90]; such issues were associated with failure in PR.

The selection of patients for rehabilitation is of paramount importance because specific characteristics may be associated with a greater response to PR. Subjects with a higher FVC, less exercise desaturation, less disability, and more severe breathlessness demonstrated a more remarkable improvement in rehabilitation outcomes [28].

The pulmonary rehabilitation program should consist of a comprehensive intervention of patient-tailored therapies [91]. In particular, exercise training is a critical component of pulmonary rehabilitation. Currently, principles of exercise training in IPF are similar to those in chronic obstructive pulmonary disease (COPD), but patients with IPF may need a modification of exercise prescription compared to COPD patients, due to the severity of dyspnea and more severe oxyhemoglobin desaturation on exertion. Nutritional assessment and support are other essential components of rehabilitation programs, which have as a principal target the recovery of skeletal muscle function. Indeed, there is evidence that a combination of both exercise training and nutrition care management may improve rehabilitation outcomes [92].

The other components of PR include education, psychological support, and training in behaviors (i.e., behaviors that affect patients’ life such as smoking, sedentary lifestyle, etc.) that will assist in disease management.

Morisset et al. investigated educational needs of IPF patients and provided some topics for educational programs such as disease education, symptoms management, medications use, oxygen therapy, and end-of-life counseling [93].

Psychological support is another component of rehabilitation programs since depression and anxiety are frequent comorbidities in IPF patients. Uncontrolled data suggest that pulmonary rehabilitation might improve psychological health in patients with IPF through improvements in symptoms such as dyspnea, exercise tolerance, and sense of control over the disease [94].

All of these components of PR must work together to achieve optimal outcomes for patients affected by IPF [95]. Studies on PR programs in IPF patient comprehensive of all the previously cited aspects are most needed.

### 3.9. Future Application and Research Perspectives

To date, evaluation of nutritional status and physical fitness, the nutrition care process, and physical activity are all largely unexplored issues related to the clinical management of patients with IPF.

The increased interest in body composition and in the assessment of nutritional status in COPD over the last few years has led to the establishment of different nutritional risk profiles, based on different patients’ metabolic phenotypes (Table 3). Such assessment proved useful in both clinical studies and patient counselling [3].

Recently, more attention has been focused on comorbidities in IPF, especially from a prognostic point of view. A prediction model, named TORVAN, assessed the ability of comorbidities to improve prediction of survival in IPF patients beyond the variables included in the GAP model. The TORVAN model also suggests that GERD, pulmonary hypertension, lung cancer, valvular heart disease, and atrial arrhythmias were the comorbidities with a higher impact on IPF patients survival [96]. There has been wide debate on nutritional alterations, such as malnutrition; one wonders whether these alterations deserve to be considered as comorbidities capable of impacting prognosis of IPF patients and which are the best indicators to assess them [97].

However, epidemiological studies performing a complete nutritional evaluation (NUTRIPF study, ClinicalTrials.gov Identifier: NCT03770845) and nutritional intervention (MADIET study, ClinicalTrials.gov Identifier: NCT03539289) in IPF patients are ongoing, and their results are expected in the near future.

Finally, future studies on the lung microbiome and the gut–lung axis might be of paramount importance in that antifibrotic drugs may affect gut microbiome leading to gastrointestinal events in some patients.

## 4. Conclusions

As far as IPF is concerned, different studies [10,11,12,98] have found that nutritional abnormalities, such as lower BMI, body weight loss, and vitamin D deficiency, seem to have a negative prognostic significance. Despite this evidence, such topics have not been examined extensively.

We believe, based on literature data broadly reported in this paper, that more attention should be devoted to the identification of the malnutrition (undernutrition) and overnutrition, as well as of low muscle strength (dynapenia) and low physical performance, taking also into account that an adequate clinical management of comorbidities, particularly gastrointestinal complications, makes IPF drug treatments more feasible.

To the best of our knowledge, there is a paucity of longitudinal studies which should evaluate the prognostic impact of some comorbid conditions that affect IPF patients, such as obesity and cardiovascular diseases. Moreover, the current lack of nutritional intervention studies does not allow to assess their efficacy in undernourished or over nourished IPF patients.

Hence, the clinical guidelines for IPF management [5,61] should incorporate recommendations for nutritional abnormalities associated with this disease.

In light of the above, it seems therefore quite clear that a multidisciplinary approach is needed not only in the diagnostic process, but also in follow-up and advanced phases of the disease—so that various specialists should be involved in multidisciplinary care of IPF, including nutritionists (physicians and dietitians) and rehabilitation specialists in addition to pulmonologists, nurses, and psychologists. Such holistic care might improve IPF patients’ quality of life and at the same time may be also a clear reference point for patients, who often feel alone with their disease [99].

## Figures and Tables

**Table 1:** Mean BMI values of patients with idiopathic pulmonary fibrosis (IPF) as reported in selected studies.

	Year of Publication	Country	Type of Study	Patients	Mean BMI (kg/m^2^)	BMI Categories (BMI as kg/m^2^)
WESTERN COUNTRIES						
Alakhras	2007	USA	Cross-sectional	*n* = 197 70% men	28.2 ± 3.0	<25: 23% 25–30: 43% >30: 34%
Mura	2012	Italy	Newly diagnosed Patients	*n* = 115 62% men	28.0 ± 4.0	-
Nolan	2018	UK	Cross-sectional	*n* = 46 72% men	27.1 ± 5.8	-
Guler	2019	Canada	Cross-sectional	*n* = 115 62% men	28.0 ± 4.0	-
Jouneau	2019	France	Cross-sectional	*n* = 81 88% men	26.3 ± 3.3	<21: 3.7%
Jouneau	2019	France	Cross-sectional	*n* = 191 84% men	27.5 ± 4.0	-
Nolan	2019	UK	Cross-sectional	*n* = 130 83% men	27.8 ± 4.7	-
Sheth	2019	USA	Cross-sectional	*n* = 50 66% men	30.2 ± 4.4	>30: 46%
FAR EAST COUNTRIES						
Kim	2012	South Corea	Newly diagnosed Patients		22.7 ± 2.9	-
Morino	2017	Japan	Cross-sectional	*n* = 38 68% men	23.9 ± 3.0	-
Nishiyama	2017	Japan	Cross-sectional	*n* = 44 80% men	22.8 ± 2.9	-
Nakatsuka	2018	Japan	Cross-sectional	*n* = 124 85% men	23.8 ± 2.6	-
Ikeda	2019	Japan	Cross-sectional	*n* = 30 80% men	21.0 ± 2.0	-

**Table 2:** Nutritional and dietary indications according to the gastrointestinal adverse events.

Diarrhea	Nausea and Vomiting	Appetite Loss
Maintain good hydration with a fluid intake of at least three liters per day. Use simple cooking methods, such as steaming, microwave baking and grilling. Use raw extra-virgin olive oil to flavor foods. Eat carrot and potato soup without vegetables, initially. Fruit intake should be no more than two servings per day and always peeled. Eat legumes as creamy soup.	It is best to drink fluids before or after meals, not while eating. Eat small and frequent meals. Eat slowly and chewing food thoroughly. Eat lightly seasoned, not excessively aromatic, low-fat foods, without sauces or strong spices. Foods should be cooked through simple methods, such as grilling, roasting, baking and boiling. Eat dry foods, such as rusks, bread and biscuits. Limit or avoid drinks with caffeine because they may worsen nausea. Eat when you are hungry in order to avoid food refusal. Drink small amounts of liquid (infusions, fruit juices) frequently. Liquids and soft, slightly warm and/or cold foods may be more tolerable than hot ones: fruit sorbets, creams, ice creams, fruit jellies, fruit juices Carbonated drinks such as cola, soda and tonic water may alleviate gastrointestinal symptoms. Milk and its derivatives (yogurt, ricotta, low-fat cheeses) contribute essential nutrients to the diet. Add ginger or peppermint (spices with antiemetic and prokinetic properties) to your foods, declaring the possible concurrent use of anticoagulants to your doctor.	Eat small and frequent meals. Eat when appetite appears, not waiting for the usual meal times. Eat three main meals per day (breakfast, lunch and dinner), snacking between meals. Snacks should be constituted of high-calorie foods. Avoid drinking liquids before and/or during meals in that they may cause satiety. Eat vegetables accompanying them with high-calorie foods (cheese, eggs, chicken, meat, beans, corn) at the end of meals. Eat high-calorie foods, avoiding low-calorie foods, such as vegetables, salads and broths.

**Table 3:** Metabolic phenotypes applied in COPD.

Metabolic Phenotypes	Parameters and Cut-off Used to Identify Different Metabolic Phenotypes
CACHEXIA	BMI (body mass index) < 18.5 kg/m^2^
	Involuntary weight loss > 5% in the last 6 months
	FFMI (fat free mass index) < 17 kg/m^2^ for males/<15 kg/m^2^ for females
	SMI (skeletal muscle mass index) (12) < 8.87 kg/m^2^ for males
	/<6.42 kg/m^2^ for females
	BFMI (body fat mass index) < 1.7 kg/m^2^ for males/<3.8 kg/m^2^ for females
SARCOPENIA	BMI < 30 kg/m^2^
	FFMI < 17 kg/m^2^ for males/<15 kg/m^2^ for females
	SMI < 8.87 kg/m^2^ for males/<6.42 kg/m^2^ for females
	BFMI > 1.8 kg/m^2^ for males/>3.9 kg/m^2^ for females
	Hand Grip < 30 kg for males/<20 kg for females
	Gait Speed (4 m) (14) < 0.8 m/s
NORMAL NUTRITIONAL STATUS	BMI between 18.5 and 24.9 kg/m^2^, (overweight if BMI > 25 and <30)
	FFMI > 17 kg/m^2^ for males/>15 kg/m^2^ for females
	SMI > 8.88 kg/m^2^ for males/>6.43 kg/m^2^ for females
	BFMI between 1.8 and 5.2 kg/m^2^ for males/between 3.9 and 8.2 kg/m^2^ for females
	Hand Grip > 30 kg for males/>20 kg for females
	Gait Speed (4 m) > 0.9 m/s
	No involuntary weight loss > 5% in the last 6 months
OBESITY	BMI > 30.1 kg/m^2^
	Abdominal circumference > 102 cm for males/>88 cm for females
	FFMI > 17 kg/m^2^ for males/>15 kg/m^2^ for females
	SMI > 8.88 kg/m^2^ for males/>6.43 kg/m^2^ for females
	BFMI > 8.3 kg/m^2^ for males/>11.82 kg/m^2^ for females
	Hand Grip > 30 kg for males/>20 kg for females
	Gait Speed (4 m) > 0.9 m/s
	BMI > 30.1 kg/m^2^
SARCOPENIC OBESITY	Abdominal circumference > 102 cm for males/>88 cm for females
	FFMI < 17 kg/m^2^ for males/<15 kg/m^2^ for females
	SMI < 8.87 kg/m^2^ for males/<6.42 kg/m^2^ for females BFMI > 8.3 kg/m^2^ for males/>11.82 kg/m^2^ for females
	Hand Grip < 30 kg for males/<20 kg for females
	Gait Speed (4 m) < 0.8 m/s

## References

[1] Mekal D., Doboszynska A., Kadalska E., Swietlik E., Rudnicka L. (2015). Nutritional status in chronic obstructive pulmonary disease and systemic sclerosis: Two systemic diseases involving the respiratory system. Adv. Exp. Med. Biol..

[2] Hitzl A.P., Jorres R.A., Heinemann F., Pfeifer M., Budweiser S. (2010). Nutritional status in patients with chronic respiratory failure receiving home mechanical ventilation: Impact on survival. Clin. Nutr..

[3] Schols A.M., Ferreira I.M., Franssen F.M., Gosker H.R., Janssens W., Muscaritoli M., Pison C., Rutten-van Molken M., Slinde F., Steiner M.C. (2014). Nutritional assessment and therapy in COPD: A European Respiratory Society statement. Eur. Respir. J..

[4] Richeldi L., Collard H.R., Jones M.G. (2017). Idiopathic pulmonary fibrosis. Lancet.

[5] Raghu G., Collard H.R., Egan J.J., Martinez F.J., Behr J., Brown K.K., Colby T.V., Cordier J.F., Flaherty K.R., Lasky J.A. (2011). An official ATS/ERS/JRS/ALAT statement: Idiopathic pulmonary fibrosis: Evidence-based guidelines for diagnosis and management. Am. J. Respir. Crit. Care Med..

[6] Sgalla G., Iovene B., Calvello M., Ori M., Varone F., Richeldi L. (2018). Idiopathic pulmonary fibrosis: Pathogenesis and management. Respir. Res..

[7] Gea J., Badenes D., Balcells E. (2018). Nutritional status in patients with Idiopathic Pulmonary Fibrosis. Pulm. Crit. Care Med..

[8] Guler S.A., Hur S.A., Lear S.A., Camp P.G., Ryerson C.J. (2019). Body composition, muscle function, and physical performance in fibrotic interstitial lung disease: A prospective cohort study. Respir. Res..

[9] Mousavi S.E., Amini H., Heydarpour P., Amini Chermahini F., Godderis L. (2019). Air pollution, environmental chemicals, and smoking may trigger vitamin D deficiency: Evidence and potential mechanisms. Environ. Int..

[10] Alakhras M., Decker P.A., Nadrous H.F., Collazo-Clavell M., Ryu J.H. (2007). Body mass index and mortality in patients with idiopathic pulmonary fibrosis. Chest.

[11] Nakatsuka Y., Handa T., Kokosi M., Tanizawa K., Puglisi S., Jacob J., Sokai A., Ikezoe K., Kanatani K.T., Kubo T. (2018). The Clinical Significance of Body Weight Loss in Idiopathic Pulmonary Fibrosis Patients. Respiration.

[12] Pugashetti J., Graham J., Boctor N., Mendez C., Foster E., Juarez M., Harper R., Morrissey B., Kadoch M., Oldham J.M. (2018). Weight loss as a predictor of mortality in patients with interstitial lung disease. Eur. Respir. J..

[13] Cederholm T., Barazzoni R., Austin P., Ballmer P., Biolo G., Bischoff S.C., Compher C., Correia I., Higashiguchi T., Holst M. (2017). ESPEN guidelines on definitions and terminology of clinical nutrition. Clin. Nutr..

[14] Miyake Y., Sasaki S., Yokoyama T., Chida K., Azuma A., Suda T., Kudoh S., Sakamoto N., Okamoto K., Kobashi G. (2006). Dietary fat and meat intake and idiopathic pulmonary fibrosis: A case-control study in Japan. Int. J. Tuberc. Lung Dis..

[15] Miyake Y., Sasaki S., Yokoyama T., Chida K., Azuma A., Suda T., Kudoh S., Sakamoto N., Okamoto K., Kobashi G. (2004). Vegetable, fruit, and cereal intake and risk of idiopathic pulmonary fibrosis in Japan. Ann. Nutr. Metab..

[16] Mura M., Porretta M.A., Bargagli E., Sergiacomi G., Zompatori M., Sverzellati N., Taglieri A., Mezzasalma F., Rottoli P., Saltini C. (2012). Predicting survival in newly diagnosed idiopathic pulmonary fibrosis: A 3-year prospective study. Eur. Respir. J..

[17] Nolan C.M., Maddocks M., Maher T.M., Banya W., Patel S., Barker R.E., Jones S.E., George P.M., Cullinan P., Man W.D. (2019). Gait speed and prognosis in patients with idiopathic pulmonary fibrosis: A prospective cohort study. Eur. Respir. J..

[18] Jouneau S., Kerjouan M., Rousseau C., Lederlin M., Llamas-Guttierez F., De Latour B., Guillot S., Vernhet L., Desrues B., Thibault R. (2019). What are the best indicators to assess malnutrition in idiopathic pulmonary fibrosis patients? A cross-sectional study in a referral center. Nutrition.

[19] Jouneau S., Gamez A.S., Traclet J., Nunes H., Marchand-Adam S., Kessler R., Israel-Biet D., Borie R., Strombom I., Scalori A. (2019). A 2-Year Observational Study in Patients Suffering from Idiopathic Pulmonary Fibrosis and Treated with Pirfenidone: A French Ancillary Study of PASSPORT. Respiration.

[20] Nolan C.M., Maddocks M., Maher T.M., Canavan J.L., Jones S.E., Barker R.E., Patel S., Jacob J., Cullinan P., Man W.D. (2018). Phenotypic characteristics associated with slow gait speed in idiopathic pulmonary fibrosis. Respirology.

[21] Sheth J.S., Xia M., Murray S., Martinez C.H., Meldrum C.A., Belloli E.A., Salisbury M.L., White E.S., Holtze C.H., Flaherty K.R. (2019). Frailty and geriatric conditions in older patients with idiopathic pulmonary fibrosis. Respir Med..

[22] Kim J.H., Lee J.H., Ryu Y.J., Chang J.H. (2012). Clinical predictors of survival in idiopathic pulmonary fibrosis. Tuberc. Respir. Dis. (Seoul).

[23] Morino A., Takahashi H., Chiba H., Ishiai S. (2017). Daily physical activity affects exercise capacity in patients with idiopathic pulmonary fibrosis. J. Phys. Ther. Sci..

[24] Nishiyama O., Yamazaki R., Sano H., Iwanaga T., Higashimoto Y., Kume H., Tohda Y. (2017). Fat-free mass index predicts survival in patients with idiopathic pulmonary fibrosis. Respirology.

[25] Ikeda S., Sekine A., Baba T., Katano T., Tabata E., Shintani R., Sadoyama S., Yamakawa H., Oda T., Okuda R. (2019). Negative impact of anorexia and weight loss during prior pirfenidone administration on subsequent nintedanib treatment in patients with idiopathic pulmonary fibrosis. BMC Pulm. Med..

[26] Tzilas V., Bouros E., Barbayianni I., Karampitsakos T., Kourtidou S., Ntassiou M., Ninou I., Aidinis V., Bouros D., Tzouvelekis A. (2019). Vitamin D prevents experimental lung fibrosis and predicts survival in patients with idiopathic pulmonary fibrosis. Pulm. Pharmacol. Ther..

[27] Cruz-Jentoft A.J., Bahat G., Bauer J., Boirie Y., Bruyere O., Cederholm T., Cooper C., Landi F., Rolland Y., Sayer A.A. (2019). Sarcopenia: Revised European consensus on definition and diagnosis. Age Ageing.

[28] Kozu R., Senjyu H., Jenkins S.C., Mukae H., Sakamoto N., Kohno S. (2011). Differences in response to pulmonary rehabilitation in idiopathic pulmonary fibrosis and chronic obstructive pulmonary disease. Respiration.

[29] Lancaster L.H. (2018). Utility of the six-minute walk test in patients with idiopathic pulmonary fibrosis. Multidiscip. Respir. Med..

[30] Prescott E., Almdal T., Mikkelsen K.L., Tofteng C.L., Vestbo J., Lange P. (2002). Prognostic value of weight change in chronic obstructive pulmonary disease: Results from the Copenhagen City Heart Study. Eur. Respir J..

[31] Chang S.H., McDonald S.P. (2008). Post-kidney transplant weight change as marker of poor survival outcomes. Transplantation.

[32] American Thoracic Society (2000). Idiopathic pulmonary fibrosis: Diagnosis and treatment. International consensus statement. American Thoracic Society (ATS), and the European Respiratory Society (ERS). Am. J. Respir Crit Care Med..

[33] Bahmer T., Kirsten A.M., Waschki B., Rabe K.F., Magnussen H., Kirsten D., Gramm M., Hummler S., Brunnemer E., Kreuter M. (2016). Clinical Correlates of Reduced Physical Activity in Idiopathic Pulmonary Fibrosis. Respiration.

[34] Suzuki Y., Yoshimura K., Enomoto Y., Yasui H., Hozumi H., Karayama M., Furuhashi K., Enomoto N., Fujisawa T., Nakamura Y. (2018). Distinct profile and prognostic impact of body composition changes in idiopathic pulmonary fibrosis and idiopathic pleuroparenchymal fibroelastosis. Sci. Rep..

[35] Collard H.R., Ryerson C.J., Corte T.J., Jenkins G., Kondoh Y., Lederer D.J., Lee J.S., Maher T.M., Wells A.U., Antoniou K.M. (2016). Acute Exacerbation of Idiopathic Pulmonary Fibrosis. An International Working Group Report. Am. J. Respir. Crit. Care Med..

[36] Sungurtekin H., Sungurtekin U., Oner O., Okke D. (2008). Nutrition assessment in critically ill patients. Nutr. Clin. Pract..

[37] Martin C.M., Doig G.S., Heyland D.K., Morrison T., Sibbald W.J. (2004). Southwestern Ontario Critical Care Research Network. Multicentre, cluster-randomized clinical trial of algorithms for critical-care enteral and parenteral therapy (ACCEPT). CMAJ.

[38] Rodriguez L. (2004). Nutritional status: Assessing and understanding its value in the critical care setting. Crit. Care Nurs. Clin. North. Am..

[39] Krishnan J.A., Parce P.B., Martinez A., Diette G.B., Brower R.G. (2003). Caloric intake in medical ICU patients: Consistency of care with guidelines and relationship to clinical outcomes. Chest.

[40] Jolliet P., Pichard C., Biolo G., Chiolero R., Grimble G., Leverve X., Nitenberg G., Novak I., Planas M., Preiser J.C. (1999). Enteral nutrition in intensive care patients: A practical approach. Clin. Nutr..

[41] Huang Y.C., Yen C.E., Cheng C.H., Jih K.S., Kan M.N. (2000). Nutritional status of mechanically ventilated critically ill patients: Comparison of different types of nutritional support. Clin. Nutr..

[42] Thomas J.M., Isenring E., Kellett E. (2007). Nutritional status and length of stay in patients admitted to an Acute Assessment Unit. J. Hum. Nutr. Diet..

[43] Kizer J.R., Zisman D.A., Blumenthal N.P., Kotloff R.M., Kimmel S.E., Strieter R.M., Arcasoy S.M., Ferrari V.A., Hansen-Flaschen J. (2004). Association between pulmonary fibrosis and coronary artery disease. Arch. Intern. Med..

[44] Nathan S.D., Basavaraj A., Reichner C., Shlobin O.A., Ahmad S., Kiernan J., Burton N., Barnett S.D. (2010). Prevalence and impact of coronary artery disease in idiopathic pulmonary fibrosis. Respir. Med..

[45] Faverio P., De Giacomi F., Bonaiti G., Stainer A., Sardella L., Pellegrino G., Sferrazza Papa G.F., Bini F., Bodini B.D., Carone M. (2019). Management of Chronic Respiratory Failure in Interstitial Lung Diseases: Overview and Clinical Insights. Int. J. Med. Sci..

[46] Oldham J.M., Collard H.R. (2017). Comorbid Conditions in Idiopathic Pulmonary Fibrosis: Recognition and Management. Front. Med. (Lausanne).

[47] Wernig G., Chen S.Y., Cui L., Van Neste C., Tsai J.M., Kambham N., Vogel H., Natkunam Y., Gilliland D.G., Nolan G. (2017). Unifying mechanism for different fibrotic diseases. Proc. Natl. Acad. Sci. USA.

[48] Kreuter M., Lederer D.J., Molina-Molina M., Noth I., Valenzuela C., Frankenstein L., Weycker D., Atwood M., Kirchgaessler K.U., Cottin V. (2019). Association of Angiotensin Modulators With the Course of Idiopathic Pulmonary Fibrosis. Chest.

[49] Kreuter M., Bonella F., Maher T.M., Costabel U., Spagnolo P., Weycker D., Kirchgaessler K.U., Kolb M. (2017). Effect of statins on disease-related outcomes in patients with idiopathic pulmonary fibrosis. Thorax.

[50] Kheirollahi V., Wasnick R.M., Biasin V., Vazquez-Armendariz A.I., Chu X., Moiseenko A., Weiss A., Wilhelm J., Zhang J.S., Kwapiszewska G. (2019). Metformin induces lipogenic differentiation in myofibroblasts to reverse lung fibrosis. Nat. Commun..

[51] Rangarajan S., Bone N.B., Zmijewska A.A., Jiang S., Park D.W., Bernard K., Locy M.L., Ravi S., Deshane J., Mannon R.B. (2018). Metformin reverses established lung fibrosis in a bleomycin model. Nat. Med..

[52] Sato N., Takasaka N., Yoshida M., Tsubouchi K., Minagawa S., Araya J., Saito N., Fujita Y., Kurita Y., Kobayashi K. (2016). Metformin attenuates lung fibrosis development via NOX4 suppression. Respir. Res..

[53] Spagnolo P., Kreuter M., Maher T.M., Wuyts W., Bonella F., Corte T.J., Kopf S., Weycker D., Kirchgaessler K.U., Ryerson C.J. (2018). Metformin Does Not Affect Clinically Relevant Outcomes in Patients with Idiopathic Pulmonary Fibrosis. Respiration.

[54] Gries C.J., Bhadriraju S., Edelman J.D., Goss C.H., Raghu G., Mulligan M.S. (2015). Obese patients with idiopathic pulmonary fibrosis have a higher 90-day mortality risk with bilateral lung transplantation. J. Heart Lung Transplant..

[55] Oldham J.M., Kumar D., Lee C., Patel S.B., Takahashi-Manns S., Demchuk C., Strek M.E., Noth I. (2015). Thyroid Disease Is Prevalent and Predicts Survival in Patients With Idiopathic Pulmonary Fibrosis. Chest.

[56] Bedard Methot D., Leblanc E., Lacasse Y. (2019). Meta-analysis of Gastroesophageal Reflux Disease and Idiopathic Pulmonary Fibrosis. Chest.

[57] Vakil N., van Zanten S.V., Kahrilas P., Dent J., Jones R. (2006). Global Consensus Group. The Montreal definition and classification of gastroesophageal reflux disease: A global evidence-based consensus. Am. J. Gastroenterol..

[58] Katz P.O., Gerson L.B., Vela M.F. (2013). Guidelines for the diagnosis and management of gastroesophageal reflux disease. Am. J. Gastroenterol..

[59] Bolier E.A., Kessing B.F., Smout A.J., Bredenoord A.J. (2015). Systematic review: Questionnaires for assessment of gastroesophageal reflux disease. Dis. Esophagus.

[60] Gyawali C.P., Kahrilas P.J., Savarino E., Zerbib F., Mion F., Smout A.J.P.M., Vaezi M., Sifrim D., Fox M.R., Vela M.F. (2018). Modern diagnosis of GERD: The Lyon Consensus. Gut.

[61] Xaubet A., Molina-Molina M., Acosta O., Bollo E., Castillo D., Fernandez-Fabrellas E., Rodriguez-Portal J.A., Valenzuela C., Ancochea J. (2017). Guidelines for the medical treatment of idiopathic pulmonary fibrosis. Arch. Bronconeumol..

[62] Behr J., Gunther A., Bonella F., Geissler K., Koschel D., Kreuter M., Prasse A., Schonfeld N., Sitter H., Muller-Quernheim J. (2018). German Guideline for Idiopathic Pulmonary Fibrosis - Update on Pharmacological Therapies 2017. Pneumologie.

[63] Raghu G., Rochwerg B., Zhang Y., Garcia C.A., Azuma A., Behr J., Brozek J.L., Collard H.R., Cunningham W., Homma S. (2015). An Official ATS/ERS/JRS/ALAT Clinical Practice Guideline: Treatment of Idiopathic Pulmonary Fibrosis. An Update of the 2011 Clinical Practice Guideline. Am. J. Respir. Crit. Care Med..

[64] Lambert A.A., Lam J.O., Paik J.J., Ugarte-Gil C., Drummond M.B., Crowell T.A. (2015). Risk of community-acquired pneumonia with outpatient proton-pump inhibitor therapy: A systematic review and meta-analysis. PLOS ONE.

[65] Newberry C., Lynch K. (2019). The role of diet in the development and management of gastroesophageal reflux disease: Why we feel the burn. J Thorac. Dis..

[66] Bharadwaj S., Tandon P., Gohel T., Corrigan M.L., Coughlin K.L., Shatnawei A., Chatterjee S., Kirby D.F. (2015). Gastrointestinal Manifestations, Malnutrition, and Role of Enteral and Parenteral Nutrition in Patients with Scleroderma. J. Clin. Gastroenterol..

[67] Kishaba T., Nagano H., Nei Y., Yamashiro S. (2016). Body mass index-percent forced vital capacity-respiratory hospitalization: New staging for idiopathic pulmonary fibrosis patients. J. Thorac. Dis..

[68] Kulkarni T., Yuan K., Tran-Nguyen T.K., Kim Y.I., de Andrade J.A., Luckhardt T., Valentine V.G., Kass D.J., Duncan S.R. (2019). Decrements of body mass index are associated with poor outcomes of idiopathic pulmonary fibrosis patients. PLoS ONE.

[69] Perelas A., Glennie J., van Kerkhove K., Li M., Scheraga R.G., Olman M.A., Culver D.A. (2019). Choice of antifibrotic medication and disease severity predict weight loss in idiopathic pulmonary fibrosis. Pulm. Pharmacol. Ther..

[70] Corte T., Bonella F., Crestani B., Demedts M.G., Richeldi L., Coeck C., Pelling K., Quaresma M., Lasky J.A. (2015). Safety, tolerability and appropriate use of nintedanib in idiopathic pulmonary fibrosis. Respir. Res..

[71] Crestani B., Huggins J.T., Kaye M., Costabel U., Glaspole I., Ogura T., Song J.W., Stansen W., Quaresma M., Stowasser S. (2019). Long-term safety tolerability of nintedanib in patients with idiopathic pulmonary fibrosis: Results from the open-label extension study, INPULSIS-ON. Lancet Respir. Med..

[72] Noth I., Oelberg D., Kaul M., Conoscenti C.S., Raghu G. (2018). Safety and tolerability of nintedanib in patients with idiopathic pulmonary fibrosis in the USA. Eur. Respir. J..

[73] Brunnemer E., Walscher J., Tenenbaum S., Hausmanns J., Schulze K., Seiter M., Heussel C.P., Warth A., Herth F.J.F., Kreuter M. (2018). Real-World Experience with Nintedanib in Patients with Idiopathic Pulmonary Fibrosis. Respiration.

[74] Bendstrup E., Wuyts W., Alfaro T., Chaudhuri N., Cornelissen R., Kreuter M., Melgaard Nielsen K., Munster A.B., Myllarniemi M., Ravaglia C. (2019). Nintedanib in Idiopathic Pulmonary Fibrosis: Practical Management Recommendations for Potential Adverse Events. Respiration.

[75] Ikeda S., Sekine A., Baba T., Yamanaka Y., Sadoyama S., Yamakawa H., Oda T., Okuda R., Kitamura H., Okudela K. (2017). Low body surface area predicts hepatotoxicity of nintedanib in patients with idiopathic pulmonary fibrosis. Sci. Rep..

[76] Lancaster L., Albera C., Bradford W.Z., Costabel U., du Bois R.M., Fagan E.A., Fishman R.S., Glaspole I., Glassberg M.K., King T.E. (2016). Safety of pirfenidone in patients with idiopathic pulmonary fibrosis: Integrated analysis of cumulative data from 5 clinical trials. BMJ Open Respir. Res..

[77] Nathan S.D., Costabel U., Albera C., Behr J., Wuyts W.A., Kirchgaessler K.U., Stauffer J.L., Morgenthien E., Chou W., Limb S.L. (2019). Pirfenidone in patients with idiopathic pulmonary fibrosis and more advanced lung function impairment. Respir. Med..

[78] Costabel U., Albera C., Glassberg M.K., Lancaster L.H., Wuyts W.A., Petzinger U., Gilberg F., Kirchgaessler K.U., Noble P.W. (2019). Effect of pirfenidone in patients with more advanced idiopathic pulmonary fibrosis. Respir. Res..

[79] Yoon H.Y., Kim D.S., Song J.W. (2019). Efficacy and Safety of Pirfenidone in Advanced Idiopathic Pulmonary Fibrosis. Respiration.

[80] Barratt S.L., Mulholland S., Al Jbour K., Steer H., Gutsche M., Foley N., Srivastava R., Sharp C., Adamali H.I. (2018). South-West of England's Experience of the Safety and Tolerability Pirfenidone and Nintedanib for the Treatment of Idiopathic Pulmonary Fibrosis (IPF). Front. Pharmacol..

[81] Caro F.M., Alberti M.L., Campins F., Enghelmayer J.I., Fernandez M.E., Lancellotti D., Papucci T., Sebastiani J.A., Paulin F. (2019). Real-Life Experience with Pirfenidone in Idiopathic Pulmonary Fibrosis in Argentina. A Retrospective Multicenter Study. Arch. Bronconeumol..

[82] Margaritopoulos G.A., Trachalaki A., Wells A.U., Vasarmidi E., Bibaki E., Papastratigakis G., Detorakis S., Tzanakis N., Antoniou K.M. (2018). Pirfenidone improves survival in IPF: Results from a real-life study. BMC Pulm. Med..

[83] Tzouvelekis A., Karampitsakos T., Ntolios P., Tzilas V., Bouros E., Markozannes E., Malliou I., Anagnostopoulos A., Granitsas A., Steiropoulos P. (2017). Longitudinal “Real-World” Outcomes of Pirfenidone in Idiopathic Pulmonary Fibrosis in Greece. Front. Med. (Lausanne).

[84] Nathan S.D., Lancaster L.H., Albera C., Glassberg M.K., Swigris J.J., Gilberg F., Kirchgaessler K.U., Limb S.L., Petzinger U., Noble P.W. (2018). Dose modification and dose intensity during treatment with pirfenidone: Analysis of pooled data from three multinational phase III trials. BMJ Open Respir. Res..

[85] Flaherty K.R., Fell C.D., Huggins J.T., Nunes H., Sussman R., Valenzuela C., Petzinger U., Stauffer J.L., Gilberg F., Bengus M. (2018). Safety of nintedanib added to pirfenidone treatment for idiopathic pulmonary fibrosis. Eur. Respir. J..

[86] Wadler S., Benson A.B., Engelking C., Catalano R., Field M., Kornblau S.M., Mitchell E., Rubin J., Trotta P., Vokes E. (1998). Recommended guidelines for the treatment of chemotherapy-induced diarrhea. J. Clin. Oncol..

[87] Yu X., Li X., Wang L., Liu R., Xie Y., Li S., Li J. (2019). Pulmonary Rehabilitation for Exercise Tolerance and Quality of Life in IPF Patients: A Systematic Review and Meta-Analysis. Biomed. Res. Int..

[88] Perez-Bogerd S., Wuyts W., Barbier V., Demeyer H., Van Muylem A., Janssens W., Troosters T. (2018). Short and long-term effects of pulmonary rehabilitation in interstitial lung diseases: A randomised controlled trial. Respir Res..

[89] Dowman L.M., McDonald C.F., Hill C.J., Lee A.L., Barker K., Boote C., Glaspole I., Goh N.S.L., Southcott A.M., Burge A.T. (2017). The evidence of benefits of exercise training in interstitial lung disease: A randomised controlled trial. Thorax.

[90] Chehere B., Bougault V., Chenivesse C., Grosbois J.M., Wallaert B. (2019). Cardiorespiratory adaptation during 6-Minute Walk Test in fibrotic idiopathic interstitial pneumonia patients who did or did not respond to pulmonary rehabilitation. Eur J. Phys Rehabil Med..

[91] Spruit M.A., Singh S.J., Garvey C., ZuWallack R., Nici L., Rochester C., Hill K., Holland A.E., Lareau S.C., Man W.D. (2013). An official American Thoracic Society/European Respiratory Society statement: Key concepts and advances in pulmonary rehabilitation. Am. J. Respir Crit. Care Med..

[92] Wakabayashi H., Sakuma K. (2014). Rehabilitation nutrition for sarcopenia with disability: A combination of both rehabilitation and nutrition care management. J. Cachexia Sarcopenia Muscle.

[93] Morisset J., Dube B.P., Garvey C., Bourbeau J., Collard H.R., Swigris J.J., Lee J.S. (2016). The Unmet Educational Needs of Patients with Interstitial Lung Disease. Setting the Stage for Tailored Pulmonary Rehabilitation. Ann. Am. Thorac. Soc..

[94] Ryerson C.J., Cayou C., Topp F., Hilling L., Camp P.G., Wilcox P.G., Khalil N., Collard H.R., Garvey C. (2014). Pulmonary rehabilitation improves long-term outcomes in interstitial lung disease: A prospective cohort study. Respir. Med..

[95] Nakazawa A., Cox N.S., Holland A.E. (2017). Current best practice in rehabilitation in interstitial lung disease. Ther. Adv. Respir. Dis..

[96] Torrisi S.E., Ley B., Kreuter M., Wijsenbeek M., Vittinghoff E., Collard H.R., Vancheri C. (2019). The added value of comorbidities in predicting survival in idiopathic pulmonary fibrosis: A multicentre observational study. Eur. Respir. J..

[97] Jouneau S., Lederlin M., Vernhet L., Thibault R. (2019). Malnutrition in idiopathic pulmonary fibrosis: The great forgotten comorbidity!. Eur. Respir. J..

[98] Ley B., Ryerson C.J., Vittinghoff E., Ryu J.H., Tomassetti S., Lee J.S., Poletti V., Buccioli M., Elicker B.M., Jones K.D. (2012). A multidimensional index and staging system for idiopathic pulmonary fibrosis. Ann. Intern. Med..

[99] Prades J., Remue E., van Hoof E., Borras J.M. (2015). Is it worth reorganising cancer services on the basis of multidisciplinary teams (MDTs)? A systematic review of the objectives and organisation of MDTs and their impact on patient outcomes. Health Policy.

